# Heptamethine Cyanine–Based Application for Cancer Theranostics

**DOI:** 10.3389/fphar.2021.764654

**Published:** 2022-02-11

**Authors:** Lei Zhang, Hang Jia, Xuqian Liu, Yaxin Zou, Jiayi Sun, Mengyu Liu, Shuangshuang Jia, Nan Liu, Yanzhang Li, Qun Wang

**Affiliations:** ^1^ School of Basic Medical Sciences, Laboratory for Nanomedicine, Henan University, Kaifeng, China; ^2^ School of Clinical Medicine, Henan University, Kaifeng, China; ^3^ Obstetrics Department, Kaifeng Maternity Hospital, Kaifeng, China

**Keywords:** heptamethine cyanine, cancer theranostics, NIR fluorescence imaging, phototherapy, nanoprobes

## Abstract

Cancer is the most common life-threatening malignant disease. The future of personalized cancer treatments relies on the development of functional agents that have tumor-targeted anticancer activities and can be detected in tumors through imaging. Cyanines, especially heptamethine cyanine (Cy7), have prospective application because of their excellent tumor-targeting capacity, high quantum yield, low tissue autofluorescence, long absorption wavelength, and low background interference. In this review, the application of Cy7 and its derivatives in tumors is comprehensively explored. Cy7 is enormously acknowledged in the field of non-invasive therapy that can “detect” and “kill” tumor cells *via* near-infrared fluorescence (NIRF) imaging, photothermal therapy (PTT), and photodynamic therapy (PDT). Furthermore, Cy7 is more available and has excellent properties in cancer theranostics by the presence of multifunctional nanoparticles *via* fulfilling multimodal imaging and combination therapy simultaneously. This review provides a comprehensive scope of Cy7’s application for cancer NIRF imaging, phototherapy, nanoprobe-based combination therapy in recent years. A deeper understanding of the application of imaging and treatment underlying Cy7 in cancer may provide new strategies for drug development based on cyanine. Thus, the review will lead the way to new types with optical properties and practical transformation to clinical practice.

## Introduction

According to the statistics from the American Cancer Society, the cancer mortality rates have been recently declining. However, huge estimates of 1,806,590 new cancer cases and 606,520 deaths in the United States are still predicted by 2020 (Miller et al., 2020; Zhang et al., 2020). Cancer remains a life- and health-threatening disease that should be tackled in the future (Zhang et al., 2020).

NIRF imaging offers several advantages of bioimaging and holds great promise for tumor detection. Among NIRF dyes, Cy7 has recently received extensive research interest. Traditional cyanine dyes, such as indocyanine green (ICG) as the only near-infrared (NIR) optical marker approved by the United States Food and Drug Administration for clinical use, still have these limitations: limited photostability, high plasma protein binding rate, medium fluorescence quantum yield, short blood half-life, rapid hepatic uptake, and lack of tumor-targeting specificity (Egloff-Juras et al., 2019). Compared with ICG, Cy7 and the analogs, such as IR780, IR783 (Yang et al., 2010), and IR808/MHI-148 (Yang et al., 2010), which are characterized by hardened conjugated olefins in the cyclohexyl skeleton and substituted by meso-chlorine, have higher optical stability, higher quantum yield, and lower tissue autofluorescence and therefore, have become cancer imaging probes and a targeted therapeutic tool for cancer.

Fluorescence imaging, especially NIRF, which has great advantages in safety, detection sensitivity, and resolution, has achieved early diagnosis and visualization of various cancer types due to its convenience in *in vivo* applications, thus providing a new platform for non-invasive molecular imaging (Singh et al., 2019). Owing to the excellent tumor-targeting capacity, high extinction coefficient, and relatively large Stokes shift (Sissa et al., 2019), the fluorescence of Cy7 and the derivatives can be easily detected from deep tissues to accomplish tumor imaging under the emission profiles of 700–1,000 nm (Hu et al., 2020). Song et al. synthesized FA-IR780-NP with excellent tumor-targeting and NIRF imaging capabilities. This dye achieved the best cytoreductive surgery (residuum≤1 cm), effectively delimited the boundary of ovarian tumors, and mediated PTT to effectively eradicate ovarian cancer tumors, thus providing a safe adjuvant treatment for residual lesions in surgery. Furthermore, FA-IR780-NP can also be used as a new drug delivery system in the diagnosis and treatment of ovarian cancer due to its negligible toxicity and excellent targeting ability (Li et al., 2019).

The biodegradable Cy7 and the derivatives can also generate reactive oxygen species (ROS) under a certain wavelength of radiation to induce cancer cell apoptosis and/or produce hyperthermia to ablate tumor cells during laser irradiation. These substances are widely used in PDT and PTT with excellent spatial specificity, non-invasiveness, and minimal side effects on normal tissues. Moreover, such dyes can be loaded or bound to nanoparticles to overcome the hydrophobic properties, improve the tumor-specific delivery, and participate in the combined treatment of cancer. Wang et al. used transferrin as a drug carrier in loading IR780 iodide to form Tf-IR780 NPs, which have a high binding affinity with the transferrin receptor (TfR) overexpressed in various human tumors including breast and prostate cancer. Tf-IR780 NPs can also effectively accumulate in tumor tissues and cooperate well with PTT and PDT (Wang et al., 2016). Zhang et al. studied and synthesized Gly@Cy7-Si-DOX NPs and discovered their great prospects in controllable colorectal cancer photothermal therapy combined with chemotherapy (Zhang et al., 2019a).

Cy7-based near-infrared fluorescent probes with tumor-targeting capabilities are essential for tumor imaging, diagnosis, and treatment. Traditional Cy7s, such as IR780, IR783, and MHI-148, have been confirmed to have the ability to preferentially accumulate to the tumor site, although the mechanism is not yet clear. In addition, the vast majority of Cy7s improve the tumor-targeting ability by connecting various ligands that can bind to the overexpressed receptors in the tumor microenvironment or being coated to form nanoparticles (Yi et al., 2014). Most nanoparticles accumulate passively in tumor sites *via* the EPR effect; however, they can also actively target tumors when they are covalently linked with ligands that can specifically bind to the antigens overexpressed on the surface of tumor cells (Chinen et al., 2015; Maeda, 2015). For instance, due to the specific binding of HA and CD44, the HA-IR-Pyr formed by the electrostatic interaction between positively charged IR-Pyr and negatively charged HA, preferentially accumulates in CD44-overexpressing tumors and localizes in the mitochondria after being cleaved by hyaluronidase (Thomas et al., 2017).

**FIGURE 1:**
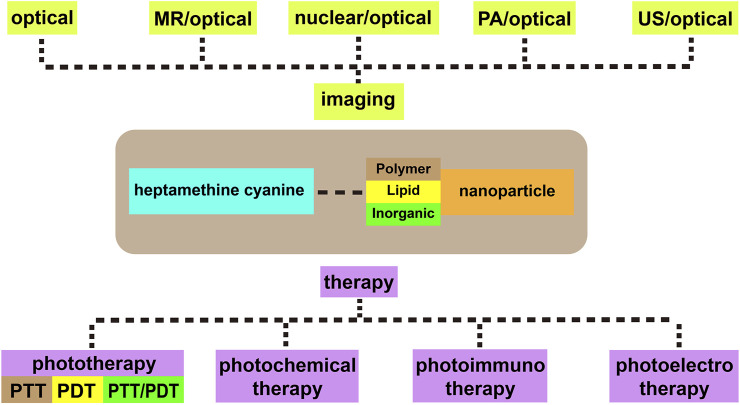
Heptamethine cyanine–based application for cancer theranostics.

This review mainly introduces the recent applications of Cy7 and its modifications in imaging, phototherapy, and combination with nanomaterials in tumors. Processing, modifying, and combining other technologies on the original structure of Cy7 make it more in-depth and extensive in tumor applications (Figure 1). For more details, information on the structure and function of Cy7 and its derivatives mentioned in this review is summarized in Table 1.

**TABLE 1:** Information on the structure and function of Cy7 and its derivatives.

Name	Structure	Function	Mechanism	Structure–activity relationship	Reference
ICG	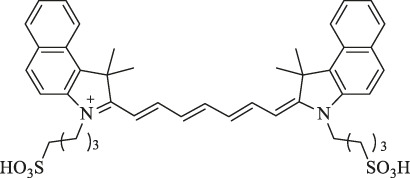	Cancer diagnosis	NIRF imaging		Egloff-Juras et al. (2019)
IR780	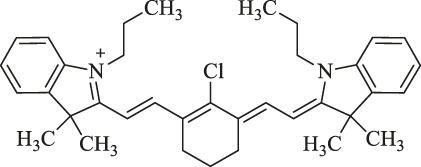	Cancer therapy	PTT		Yang et al. (2010)
IR783	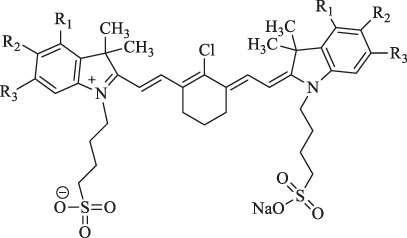	Therapy of Pancreatic cancer	PDT	Heavy atom iodine improves the singlet oxygen generation.	Atchison et al. (2017)
	6a : I2-IRCYDYE: R1, R3=H, R2=I				
	6b : I4-IRCYDYE: R1, R3=I, R2=H				
	IR783: R1=R2=R3=H				
IR808/MHI-148	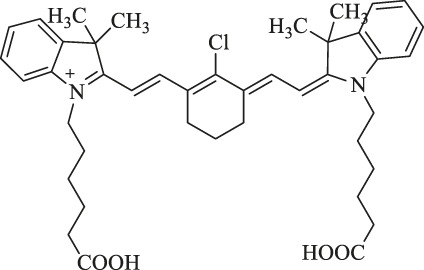	Cancer diagnosis and therapy	NIRF imaging, PDT/PTT combined treatment	Meso-chlorine improves the optical stability and quantum yield and reduces tissue autofluorescence.	Yang et al. (2010)
genistein-IR783	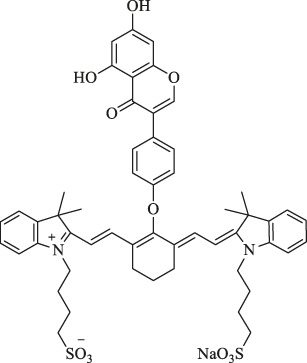	Diagnosis of breast cancer	NIRF imaging	Conjugation by covalently attaching IR783 to genistein improves the anticancer ability	Guan et al. (2019)
3-mercapto-propionic-cyclohexenyl-Cy7-bis-TMZ-CPP	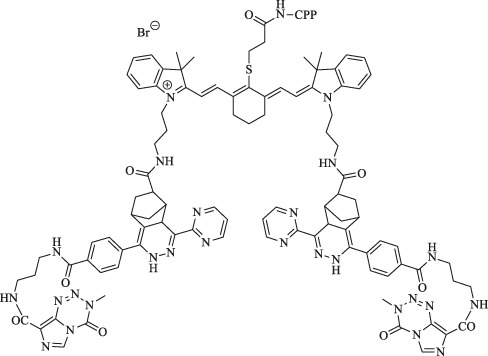	Diagnostics and therapy monitoring of cancer	NIRF imaging	CPP facilitates the formation of the molecule across cellular membranes; TMZ improves the anticancer ability	Komljenovic et al. (2016)
NIR-H_2_S probe	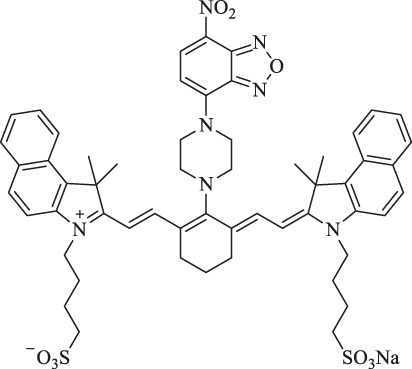	Diagnosis of breast cancer	NIRF imaging	Compounds can exhibit thiolysis reaction and then emit a fluorescence due to 7-nitro-1,2,3-benzoxadiazole (NBD) amines	Xiong et al. (2018)
pH switchable NIRF theranostic probe	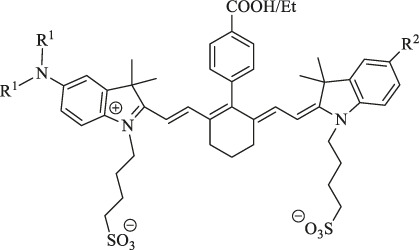	Diagnosis of liver cancer	NIRF imaging	A p-benzoic acid was substituted On the meso-position of IR783 to enhance photostability and quantum yield, an amine moiety that could donate electron was attached at the 5ʹ-position on one terminal indole ring to achieve a pH-responsive ability	Zhang J. et al. (2016)
	IR1: R_1_=H R_2_=NH_2_				
	IR2: R_1_=H R_2_=Cl				
	IR3: R_1_=H R_2_=H				
	IR4: R_1_=Et R_2_=Et_2_N				
Cy-TPP	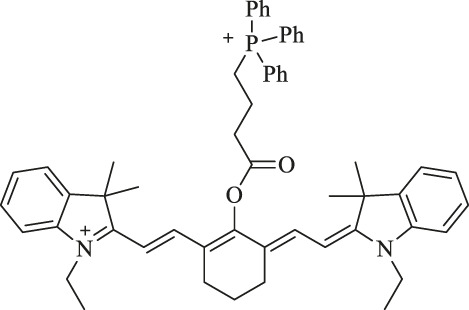	Cancer therapy and visualization		TPP moiety enhances the internalization and accumulation in the mitochondria.	Ning et al. (2017)
sorbitol-ZW800	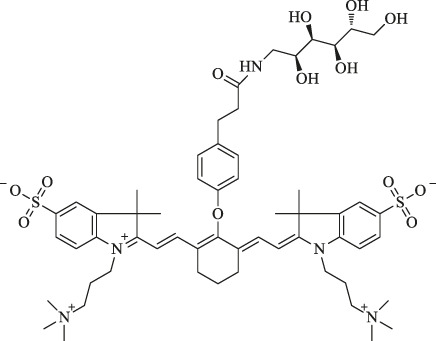	Diagnosis and therapy of colon cancer	NIRF imaging, fluorescence-guided PTT	Conjugation of sorbitol enhances the targeting ability	Lee et al. (2019)
IRDye800CW-E_2_	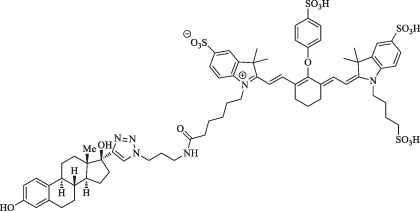	Early breast tumor detection	NIRF imaging	E_2_ analog ethinyl estradiol amine has a high affinity for ER commonly expressed at high levels in breast cancer	Tang C. et al. (2018)
ZW800-Cl	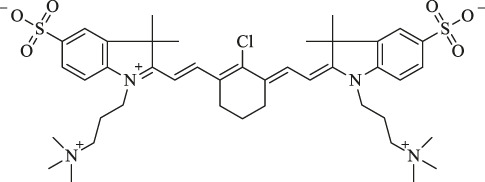	Therapy against multiple tumor types	PTT	The meso-chloride on a rigid cyclohexenyl ring enhances permeability, permeation, and the retention (EPR) effect	Lim et al. (2020)
MitDt	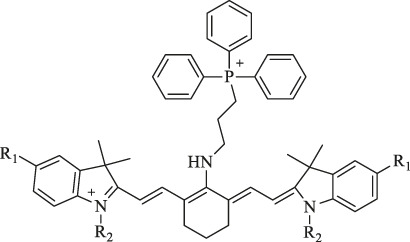	Cancer therapy	PDT	The heptamethine mesoposition is conjugated with a triphenylphosphonium derivative for mitochondrial targeting; the N-alkyl side chain is modified for regulation of charge balance and solubility, and the indolenine groups are brominated to enhance reactive oxygen species generation (ROS) after laser irradiation	Noh et al. (2018)
	1. R_1_=Br ; R_2_= 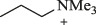	
	2. R_1_=Br ; R_2_= 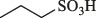	
	3. R_1_=Br ; R_2_= 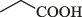	
	4. R1=H ; R2= 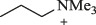	
	5. R1=H ; R2= 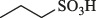	
	6. R_1_=H ; R_2_= 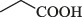
IR-Pyr	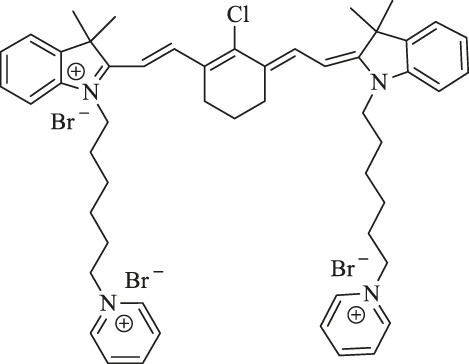	Cancer therapy	PDT	Incorporation of pyridinium ion into the indocyanine skeleton increased the water solubility	Thomas et al. (2017)
I_2_-IR783-Mpip	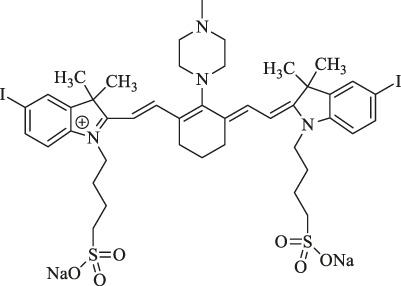	Diagnosis and therapy of liver cancer	NIRF imaging, PDT	Hcyanines were pH sensitive and produced the PDT effect.	Siriwibool et al. (2020)
DCy7	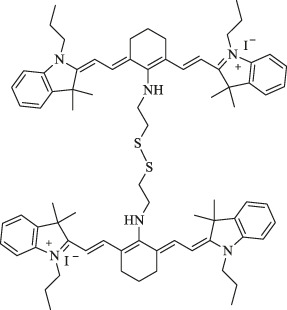	GSH-activatable pro-photosensitizer for cancer therapy	PDT		Yang G. et al. (2019)
CyI	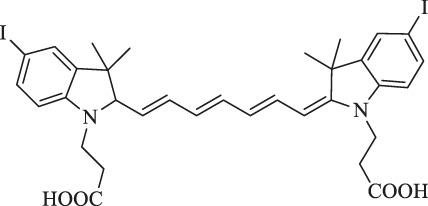	Therapy of liver cancer	PDT/PTT-combined treatment	Heavy atom iodine improves anticancer ability and induces cytotoxicity	Cao et al. (2019)
MACyanine	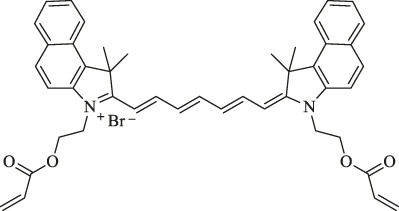	Cancer therapy	pH/NIR/heat-responsitivity	p(NIPAM-co-MACyanine-co-MCMEAM)-g-DOX with pH/NIR/heat-responsitivity	Yang R. et al. (2019)

## Heptamethine Cyanine7–Based Near-Infrared Fluorescence Imaging

As one of the most widely used optical imaging (OI) technologies, NIR imaging is a promising method for cancer diagnosis because of its high extinction coefficients (ε) and fluorescence quantum yields (Φ) at the NIR window, good biocompatibility (Zhang J. et al., 2016), radiation-less generation, high sensitivity, real-time monitoring, non-invasiveness, and low cost. It is well known that ICG has been used clinically to visualize hepatocellular carcinomas and colorectal hepatic metastases with high sensitivity (Verbeek et al., 2012). Nevertheless, the application of ICG is limited because of its instability in aqueous solutions, low fluorescence quantum yield (Leitão et al., 2020), non-specific tumor-targeting ability, concentration-dependent peak emission location, short plasma residence time, and image collection times (Fernandez-Fernandez et al., 2012; Bhattarai and Dai, 2017; Thomas and Jeong, 2017). For this reason, structurally modified Cy7 with advantages of superior tumor-targeting ability, photostability, fluorescent intensity, minimal autofluorescence (Thomas and Jeong, 2017), and low cytotoxicity provides a preferential and alternative tumor-imaging method besides the traditional ones. This review elaborates on recent application of Cy7 in tumor imaging, drug delivery and monitoring, and navigation in surgery.

### Optical Imaging Based on Heptamethine Cyanine7 Derivatives

Cy7 composed of indole heterocyclic rings, heptamethine chains, and N-substituted side chains (Sun et al., 2020) and possessing the ability of NIR absorption and mitochondria targeting is potential for cancer therapy and tumor imaging. For instance, Tan et al. identified a Cy7, IR780, which is an appealing dye for tumor-targeting imaging because of its preferential accumulation in tumor sites and high fluorescence signal with a contrast index (CI) value over 20. Inspired by this result, researchers designed and synthesized IR780 analogs, termed as IR808 or MH-148, whose CI value reached 25 *in vivo* on the 10th day and almost twice as bright as ICG, showing the latent energy as a multifunctional mitochondria-targeted agent for tumor-targeted imaging and PDT in solid tumors (e.g., kidney prostate and gastric). However, the ineligible cytotoxicity holds back its application for OI diagnosis in patients (Tan et al., 2012; Thavornpradit et al., 2019). A methylene group of IR808 was substituted with dimethyl quaternary ammonium nitrogen known as QuatCy to achieve excellent solubility and less aggregation (Thavornpradit et al., 2019). Compared with ICG, incorporating a 6-membered ring system into the linear polymethine chain minimizes non-radiative decay of IR820 *via* trans–cis isomerization, thereby increasing the fluorescence lifetimes (Lee et al., 2008a). IR820 shows higher stability and longer degradation half-times than ICG because of the structural rigidity conferred by the mesochlorobenzene but decreased quantum yields related to the chlorobenzene ring. On the basis of the prototype Cy7, the tumor-imaging property can be improved *via* structural modification or by conjugating functional moieties to the parent. Hence, fluorescent agents are conferred with versatile applications such as cancer-specific targeting, anticancer, and chemical drug–delivering *via* conjugation with tumor-targeted ligands, drug, and integration into nanomaterials. In addition, the optical imaging capacity would be enhanced as a result of specific accumulation, and pharmacokinetics may be optimized with low tissue toxicity and extended retaining time in tumors. Furthermore, the combination of NIR imaging and conventional technology compensates for each other’s limitations and thereby achieving practical clinical application. Therefore, Cy7 dyes can be efficient in changing the real-time visualization of therapeutic responses to anticancer drugs into a reality.

**FIGURE 2:**
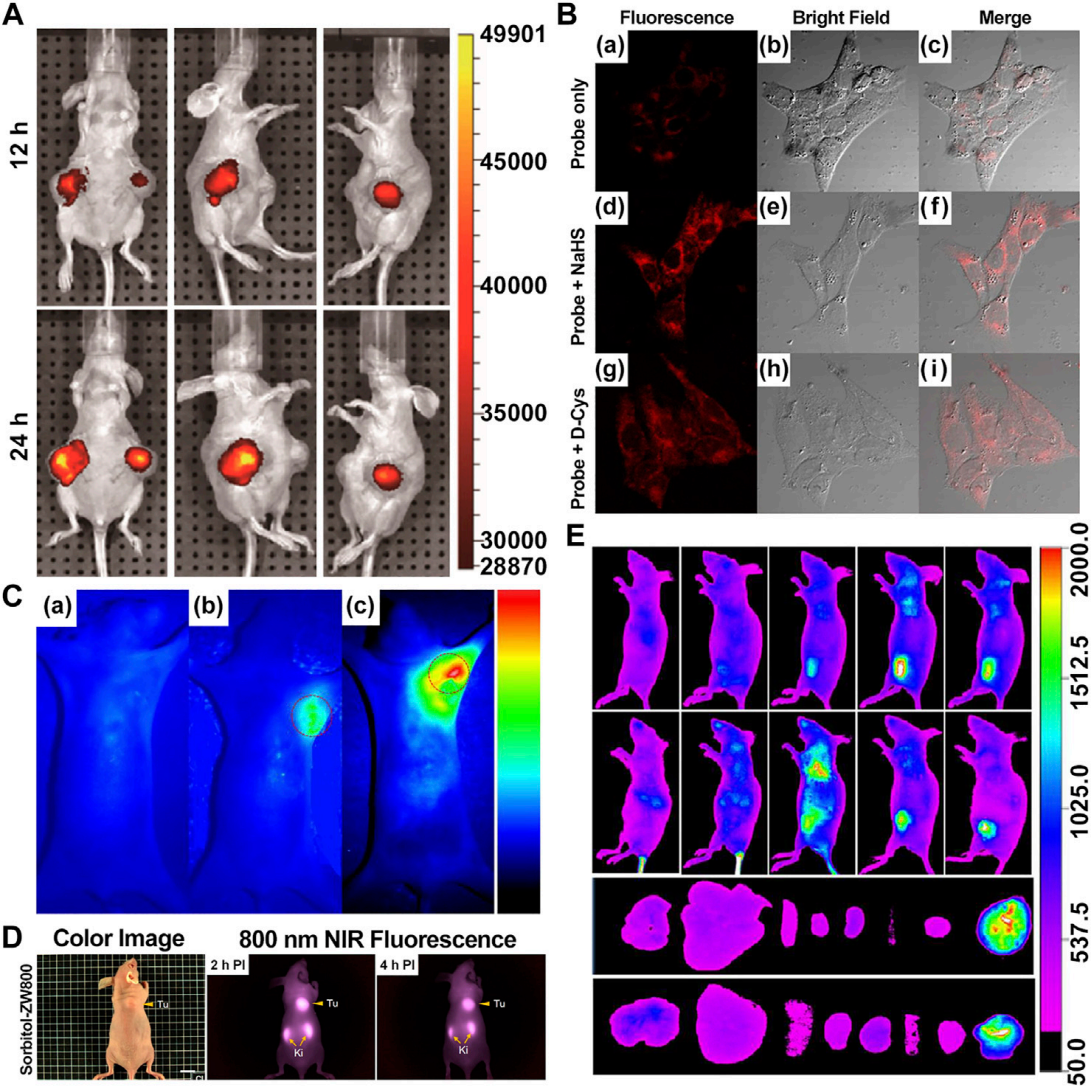
Presentation of imaging based on Cy7s. **(A)**. *In vivo* near-infrared fluorescence imaging of MCF-7 tumor-bearing mouse xenografts injected with the genistein-IR783 conjugate at 12 and 48 h, respectively. Reproduced with permission from (Guan et al., 2019). **(B)**. Fluorescence imaging of exogenous (d–f) and endogenous (g–i) H_2_S in living MCF-7 cells incubated with NIR-H_2_S for 30 min (10 μM). Reproduced with permission from (Xiong et al., 2018). **(C)**. *In vivo* NIR fluorescence images of NIR-H_2_S in 30 min after the injection: (a) Normal nude. (b) HepG2 tumor-bearing nude mouse. (c) MCF-7 tumor-bearing nude mouse. Reproduced with permission from (Xiong et al., 2018) **(D)**. *In vivo* NIR fluorescence images of HT-29 tumor-bearing mice at 2 and 4 h post-injection of sorbitol–*ZW800. Reproduced with permission from (Lee et al., 2019). **(E)**. *In vivo* tumor-targeted NIR imaging and *ex vivo* NIR images of dissected organs and tumors on A549 tumor-bearing nude mouse from 0 to 48 h after injection of IR808 and NGO-808. Reproduced with permission from (Luo et al., 2016).

Recently, Yang et al. synthesized genistein-IR783 conjugation by covalently attaching IR783, a type of Cy7, to genistein, an agent widely explored in the study of anticancer. The results showed that there was an improvement in the anticancer profile of parent genistein, tumor cell targeting, and selective uptake in the presence of IR783. *In vivo* NIR imaging revealed the presence of NIR signals in the tumor area 12 h and 2 days post-intraperitoneal injection (i.p.) (100 nM) in the MCF-7 breast cancer–bearing model, suggesting that IR783 can be a target carrier for genistein to cancer cells and thereby achieving the visualization of the anticancer activity (Figure 2A) (Guan et al., 2019).

Furthermore, 3-mercapto-propionic-cyclohexenyl-Cy7-bis-TMZ-CPP was formulated *via* multi-step chemical reactions by functionalizing the Cy7 molecule substituted with a propylene linker at indolenine-N residues with bromide cell-penetrating peptide (CPP) and temozolomide (TMZ). Based on the NIR property of Cy7, CPP facilitates the formation of the molecule across cellular membranes as an amphiphilic peptide-based module, while the effectiveness of anticancer interventions from TMZ is improved, which has a promising application in molecular cancer diagnostics and therapy monitoring (Komljenovic et al., 2016).

Xiong et al. reported that the NIR-H_2_S probe synthesized from IR820 has a clear visualization of exogenous and endogenous H_2_S in the MCF-7 human breast cancer cell at a concentration of only 10 μM. In addition, the fluorescent intensity of tumor regions in HepG2 and MCF-7 xenograft-bearing models was explicitly outstanding compared with the background at 30 min after injection (Figures 2B,C). To note, the middle product NIR-PZ was synthesized from IR820 and piperazine and then reacted with 4-chloro-7-nitrobenzofurazan to afford the probe NIR-H_2_S through the nucleophilic substitution reaction. The final product can be converted into NIR-PZ and emit a fluorescence peaked at 830 nm in the presence of H_2_S. In this regard, the fluorescence probe could be a useful candidate for H_2_S-related cancer diagnoses such as breast cancer with excellent high sensitivity, great selectivity, and low cytotoxicity profiles (Xiong et al., 2018).

The acidic extracellular microenvironment (pH 6.2–6.9) of cancer is a hallmark distinguished from normal tissues. Hence, the optical imaging of the Cy7 derivative responsive to pH is used to detect and diagnose tumors. Zhang et al. presented a class of pH-switchable NIR fluorescent theranostic probes, modified by which a p-benzoic acid was substituted on the meso-position of IR783 to enhance photostability and quantum yield (Lee et al., 2008b); an amine moiety that could donate electrons was attached at the 5ʹ-position on one terminal indole ring to achieve a pH-responsive ability. The fluorescence is regenerated due to the blocked electron transfer from the amine to Cy7 in the acid environment of lysosomes and then quenches through non-radiative decay at neutral pH. The lysosomal fluorescence signals in HepG2 and HeLa cells are higher than those in normal cells, which demonstrates that the probe only specifically illuminates cancer cells because of the acidic pH_lys_, although the delivery into lysosomes is reached in both cancer and normal cells. For this reason, the probe is hopefully used to visualize and ablate the tumor without conjugation to any cancer-targeted ligands *in vivo*, although the *in vivo* studies are ongoing (Zhang J. et al., 2016).

In order to enhance specificity in tumor imaging and increase intracellular accumulation in the region of the tumor, Cy7 is usually conjugated with receptor-targeting ligands, which can improve the fluorescent density of imaging. A novel mitochondria-targeted IR780 analog derivative Cy-TPP was synthesized by conjugating Cy7-Cl with a triphenylphosphonium (TPP) moiety. The TPP moiety is leveraged to enhance their internalization and accumulation in the mitochondria because Cy7 and TPP are lipophilic cations. Besides, the cell viability assay revealed efficient anti-proliferation due to the increased cytotoxicity from TPP, and the serum-responsive structure has stronger fluorescent intensity. Therefore, Cy-TPP could be a promising agent for cancer therapy and visualization by NIR fluorescence imaging (Ning et al., 2017).

Moreover, Lee et al. reported sorbitol–ZW800, an effective and safe NIR photothermal agent for image-guided cancer therapy, which is prepared by conjugating ZW800-1 with sorbitol used for targeting multiple types of tumor cells. The tumor tissue fluorescence signals were detected within 1 h, gradually intensified, peaked at 2 h, and finally remained at a high level for 24 h after injection in the HT-29 tumor-bearing mice model (10 nmol, 0.4 mg/kg i. v.) (Figure 2D). Therefore, it can be a potential agent in clinical tumor-specific imaging and fluorescence-guided photothermal treatment (Lee et al., 2019).

Additionally, IRDye800CW-E_2_ is likely to be brought into application for early breast tumor detection harboring high imaging sensitivity and specificity and good T/B ratios due to ERα-targeting ligand-E_2_ compared to that of sorbitol–ZW800. The fluorescence signal started to emerge at 4 h, peaked at 12 h, and lasted 48 h after injection (10 nmol i. v.) *in vivo* (Tang C. et al., 2018).

### Optical Imaging Based on Heptamethine Cyanine7 Incorporating Nanomaterials

Incorporating nanomaterials with NIR light–responsive Cy7 has been a hotspot for the diagnosis and treatment of cancer, especially for imaging. Nanostructures with well-defined physicochemical properties endow them with high passive-targeting accumulation in tumors, multiple synergistic functions, and long blood circulation retention (Cai et al., 2018; Leitão et al., 2020), thereby magnifying the tumor-imaging and tracing property of Cy7.

As mentioned above, IR780 is intrinsically equipped with favorable tumor-targeting and imaging abilities and low cytotoxicity. However, its poor hydrophobic property restricts its clinical use. Notably, IR780 phospholipid micelles show compatibility, improved pharmacokinetics, enhanced tumor accumulation, and potential NIRF signal intensity, accessible for clinical imaging of brain tumors (Li S. et al., 2017).

The MHI-HGC-PTX nanomicelle is formed by bonding hydrophobically modified glycol chitosan (HGC) to MHI-148 and then loading paclitaxel (PTX) to the nanomicelle using an oil-in-water emulsion method. MHI-148 acting as a targeting moiety and optical imaging agent prolongs the tumor retention of HGC-PTX nanomicelles and triggers PTX as an anticancer drug release. *In vivo* studies revealed that the fluorescence signals reached maximum accumulation at day 3 and lasted up to day 6 after injection (10 mg/kg i. v.) in both 4T1 and SCC7 tumor-bearing mouse models. Similarly, HGC was found to be linked with the chemical drug (Kim et al., 2006), optical imaging agent, and magnetic resonance imaging (MRI) contrast agent (Key et al., 2012) for various uses, and MHI-148 can also be incorporated with nanoparticles carrying the MRI contrast agent for NIR and MRI due to the characteristics of optical imaging and tumor targetability (Thomas et al., 2018).

CF7Ns, self-assembled nanoparticles based on chitosan modified by folate (FA) and Cy7, enhance targeting behavior toward the FA receptor overexpressed on the surface of several tumor cells including ovary, breast, kidney, liver, and lung cancer, which could be used as a FA receptor-positive tumor-targeted imaging probe in real time (Zhang et al., 2017).

NGO-808 is produced by conjugating chemically polyethylene glycol (PEG) and branched polyethylenimine (BPEI)-modified nanographene oxide (NGO) with IR808. Owing to the enhancement of tumor cellular uptake attributed to the NGO as the carrier, NGO-808 has higher efficacy in PTT from IR808 and PDT from NGO than individual IR808 and NGO. In the meanwhile, strong fluorescence signals are clearly to be visualized at tumors with their margins without intensified background interference at 48 h after intravenous injection (0.5 mg/kg i. v.) in the A549 tumor-bearing mouse model, thus providing precise imaging-guided cancer phototherapy (Figure 2E) (Luo et al., 2016).

### Multimodel Imaging Based on Heptamethine Cyanine7

Given the drawbacks of NIR imaging, the combination of Cy7-based optical imaging with other conventional imaging techniques, such as photoacoustic imaging (PAI), ultrasound imaging (United States), MRI, computed tomography (CT), single photon emission computed tomography (SPECT), and positron emission tomography (PET), is exploited to overcome NIR imaging limitations of spatial resolution, penetration depth, and tissue scattering.

MRI/NIRF dual-modality imaging provides precise and detailed information about the tumor’s location for early-stage malignant tumor diagnosis. This method combines the MR-endowed whole-body tumor localization with that of NIR imaging advantages, such as high sensitivity and real time imaging, actualizing early stage diagnosis of cancer and NIR imaging–guided resection in surgery. Gd-Cy7-PTP/RGD was introduced as a pancreatic cancer-targeted probe by covalently conjugating Cy7 with an MR contrast agent, Gd (III). Facilitated by the peptide PTP targeting plectin-1 specifically overexpressed on the surface of pancreatic ductal adenocarcinoma cells and the peptide RGD binding to the integrin widely expressed on pancreatic duct epithelial cells and angiogenesis, the bispecific molecular probe has a clinical perspective with good retention, long cycle time, and excellent specificity. *In vivo* imaging revealed that the fluorescent intensity gradually increased to the maximum from 0.5 to 4 h and remained for 24 h. Meanwhile, the T1-weighted signals in the Panc1 tumor-bearing mouse model were enhanced gradually until 8 h after injection (150 µl/mouse and 400 μg/ml i. v.) (Wang et al., 2018).

PAI is a novel method of integrating optical imaging with ultrasound (Wang and Yao, 2016) by detecting broadband acoustic waves produced by tissue heating generated in response to laser light (Weber et al., 2016; Leitão et al., 2020). PAI overcomes optical imaging limitations such as low penetration depth and resolution and has a higher optical imaging contrast, spatial resolution, less tissue scattering, and high penetration depth, achieving centimeters with the resolution at the macroscopic scale (Safe et al., 2016). Considering the level of glutathione (GSH) inside cancer cells, Li et al. synthesized a GSH-mediated turn-on nanoparticle, DHP, for fluorescence/photoacoustic imaging and PTT/chemotherapy. The DHP structure is built through the assembly of the disulfide bond–linked hydroxyethyl starch paclitaxel (HES-SS-PTX) encapsulating DiR. The fluorescence of DiR is quenched by the aggregate fluorescence quenching (ACQ) effect caused by highly concentrating in the hydrophobic core. When the disulfide bonds are cleaved by GSH in cancer cells, the PTX is released and the fluorescence switch is turned on. In addition to monitoring the drug release, DHP can also visualize the delivery in the vessel and leakage from it. The dual-model imaging for detecting tumors can complement each other with satisfying sensitivity, resolution, and penetration depth (Weber et al., 2016; Li et al., 2019).

Although NIR fluorescence imaging has such tremendous benefits for clinical image-guided therapy and diagnosis of cancer, it still suffers from insufficient quantitation and penetration depth (Gupta et al., 2014). Nuclear imaging modalities including SPECT and PET are introduced as an alternative to NIR imaging to accomplish the visualization of deep tissues and quantitative imaging with high sensitivity and low radiation (Gupta et al., 2014; Baetke et al., 2015; Stammes et al., 2016). In general, NIRF dyes must be radiolabeled and then conjugated to a chelate such as diethylenetriaminepentaacetic acid (DTPA), which is subsequently linked with a radionuclide (Stammes et al., 2016). However, there are not many related studies reported recently because of the high production cost for radionuclides. Nevertheless, other cyanine fluorescent probes integrating PET have been invented, such as the [^18/19^F] PSMA specific fluorophore by Kommidi et al. and (^111^In-Cl_3_)-HQ4 by Stammes et al. In a previous report, a PC-1001 ∕ ^68^Ga probe was fabricated by conjugating PC-1001 with ^68^Ga for PET and fluorescence imaging in the canine tumor in large live animal models (Shi et al., 2014). This dual-modality tracer was utilized by linking with a^111^In- DTPA chelate to quantitatively and systematically assess the effects of the lengths of the polymethine chain in the arginine–glycine–aspartic (RGD)–based cyanine dye on stability, photophysical properties, and imaging ability for receptor-mediated fluorescence imaging (Buckle et al., 2018).

Additionally, FA-PEG-PLGA-Ptx@ICG-Pfh NPs reported by Liu et al. achieved the combination of OI and United States (Liu et al., 2018).

## Heptamethine Cyanine7–Based Cancer Phototherapy

Phototherapy refers to the use of laser, especially the NIR laser, to irradiate the lesion area and stimulate phototherapeutic agents to kill tumor cells, including PTT and PDT (Jung et al., 2018; Nam et al., 2018; Xue et al., 2019; Mu et al., 2020). As an important aspect of the application of Cy7 in tumor therapy, the method has great application prospects in tumors because of its improved inherent specificity, reduced side effects, and precise spatial-temporal selectivity in comparison with traditional cancer treatment such as surgery, chemotherapy, or radiotherapy (Jung et al., 2018; Nam et al., 2018; Xue et al., 2019; Mu et al., 2020).

### Photothermal Therapy

As a recent hotspot of cancer treatment, PTT usually uses photothermal agents (PTAs) to generate hyperthermia from irradiated light for the thermal ablation of tumor sites and irreversibly destroy malignant tissue (Luo et al., 2016; Porcu et al., 2016; Bhattarai and Dai, 2017; Gao G. et al., 2019; Xue et al., 2019). High absorptivity PTAs in the NIR window can effectively convert NIR light energy into heat energy, cause local temperature rises (above 42°C), and promote tumor cell apoptosis and necrosis, with negligible damage to non-irradiated tissues (Gao G. et al., 2019; Lee et al., 2019). Compared with visible light, NIR has stronger penetrability. As a result, NIR-triggered PTT features high tumor ablation efficiency, minimal invasiveness, great specificity, and good penetration *in vivo* (Luo et al., 2016; Bhattarai and Dai, 2017; Gao G. et al., 2019; Lee et al., 2019). Photothermal agents play a decisive role in PTT (Li Y. et al., 2018; Lee et al., 2019). Although several types of materials, including many inorganic nanomaterials, such as carbon nanomaterials, gold nanomaterials, and metal sulfides, have been extensively developed for PTT, most are difficult to biodegrade and face unsolved biosafety problems (Li Y. et al., 2018; Lee et al., 2019; Mu et al., 2020). As alternatives, NIR Cy7 dyes have attracted general attention owing to their considerable absorption in the NIR range, accompanying thermal emission, and preferential accumulation in tumor sites (Chen et al., 2012; Lee et al., 2017; Thomas and Jeong, 2017; Lee et al., 2019).

After ICG has been approved by the FDA, some of the heptamethine derivatives, including IR820 and IR780, have been developed and are useful in NIR PTT because of their strong absorption ability and good photothermal conversion (Li et al., 2016). The photobleaching and liver metabolism limitations of these Cy7 dyes are addressed *via* a physical or chemical modification to improve their photothermal properties further. In particular, nanoplatforms, including PTT, have become a research hotspot in cancer treatment (Bhattarai and Dai, 2017; Thomas and Jeong, 2017). The combination of nanotechnology and Cy7 dyes has great potential for the targeted delivery of drugs, the improvement of bioavailability, the reduction of side effects, and the improvement of photothermal efficiency (Bhattarai and Dai, 2017; Thomas and Jeong, 2017).

**FIGURE 3:**
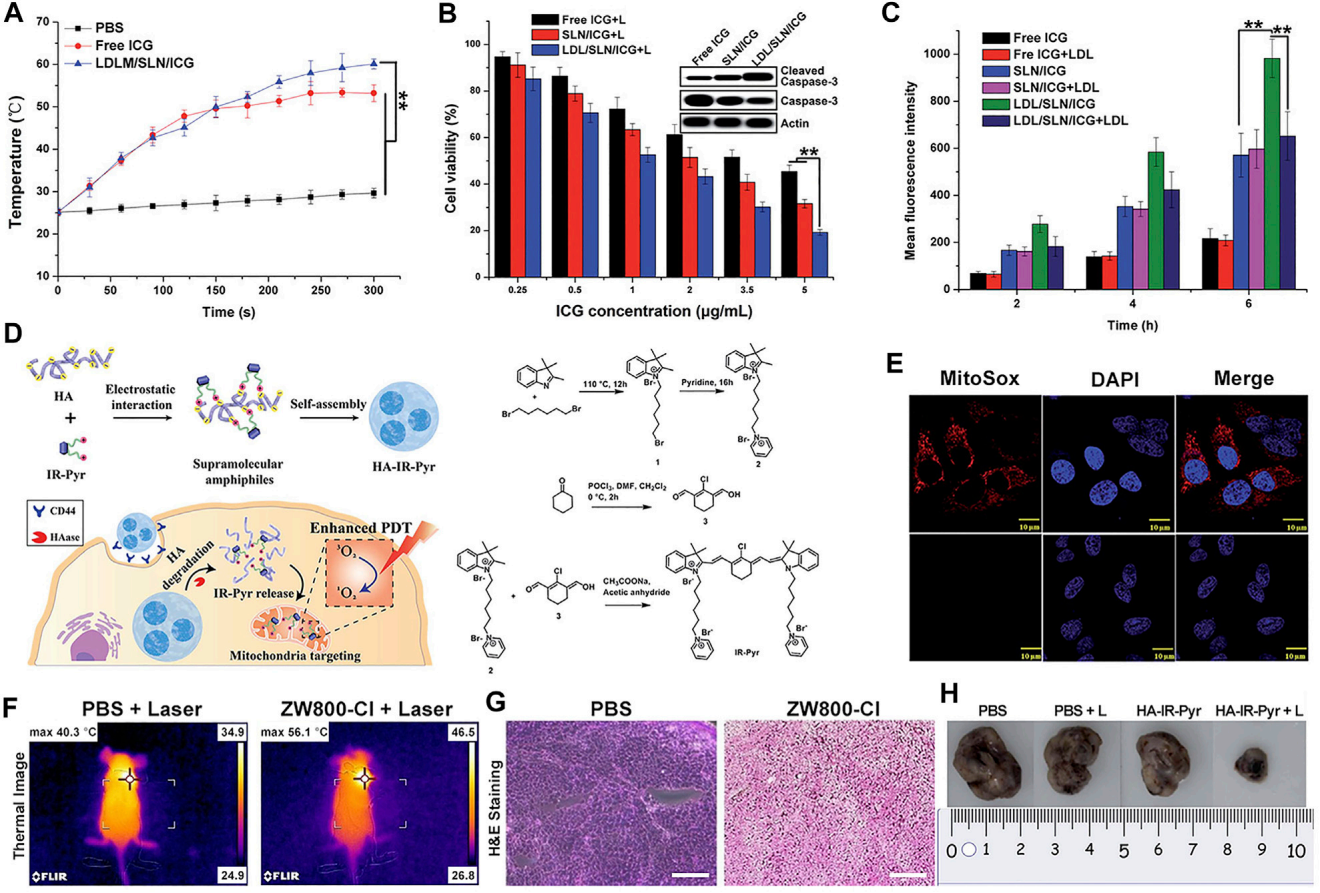
Heptamethine cyanine–based application in tumor PTT and PDT. **(A)**. Photothermal conversion of LDL/SLN/ICG, and Free ICG. Reproduced with permission from (Pan et al., 2020). **(B)**. PTT induced the anticancer effect and mechanism of free ICG, SLN/ICG, and LDL/SLN/ICG on MCF-7 cells at different ICG concentrations for 48 h under irradiation. Reproduced with permission from (Pan et al., 2020). **(C)**. Cellular accumulation of free ICG, SLN/ICG, and LDL/SLN/ICG (+LDL means pretreated with LDL) in MCF-7 cells detected by intracellular MFI. Reproduced with permission from (Pan et al., 2020). **(D)**. Schematic representation of the generation of micellar aggregates (HA-IR-Pyr), accumulation in CD44 overexpressing tumor, and tumor mitochondria localization to enhance PDT. Synthesis route of IR-Pyr. Reproduced with permission from (Thomas et al., 2017). **(E)**. Comparison of singlet oxygen generation ability in HeLa cell lines after 3 min irradiation (upper). Comparison of singlet oxygen generation ability in HeLa cell lines without irradiation (lower). Reproduced with permission from (Thomas et al., 2017). **(F)**. Tumor volume rates of each group were monitored for 7 days. Reproduced with permission from (Lim et al., 2020). **(G)**. H and E stained section of tumor of each treatment group. Reproduced with permission from (Lim et al., 2020). **(H)**. Comparison of the tumor size of each treatment group (PBS, PBS + L, HA-IR-Pyr, HA-IR-Pyr + L) after 16 days. Reproduced with permission from (Thomas et al., 2017).

ICG is a water-soluble anionic tricarbocyanine dye, and many nanocarriers have been reported to encapsulate ICG for PTT. Pan et al. prepared solid liposome nanoparticles (SLN) *via* microemulsion and encapsulated ICG inside to obtain the drug-loaded core (SLN/ICG); LDL was then used to coat SLN/ICG and finally fabricate a spherical core-shell nanoparticle (LDL/SLN/ICG) with good stability under physiological conditions. The intracellular accumulation of LDL/SLN/ICG was found to be time-dependent and greater than that of SLN/ICG in LDLR overexpressed MCF-7 cells. In addition, the anticancer efficacy of the nanoparticle was confirmed in MCF-7 cells and MCF-7 oxen-grafted Balb/c nude mice irradiated with a 1 W/cm^2^ laser (Figures 3A–C) (Pan et al., 2020).

PTT has been used against melanoma. Hwang et al. fabricated PAD hydrogels incorporated with ICG for selective attachment to melanoma and eliminate it under the NIR laser. The hydrogels were synthesized *via* the free radical polymerization of acrylamide (AM), N, N′-methylenebisacrylamide (BisAA), and diallyl dimethyl ammonium chloride (DADMAC) using a cross-linking agent. ICG can be protected against photobleaching when incorporated with PAD, thus implying PAD-ICG’s reusability. The antitumor effect of PTT was found to be highly effective with almost complete removal of the melanoma on day 10 with 808 nm laser irradiation (1 W/cm^2^) and the attachment of PAD-0.2ICG. In addition, the results showed that the mechanism of removal of melanoma cells induced by the NIR laser and PAD-ICG was apoptosis and necrosis (Hwang and Jin, 2020).

Compared with ZW800-1 disability in tumor targetability due to balanced surface charges on its structure (Lim et al., 2020), ZW800-Cl as one of ZW800-1 derivatives, was shown to be useful in targeted photothermal cancer therapy that does not require chemical modifications using photosensitizers and tumor-specific ligands. Lim et al. prepared a key intermediate ZW800-Cl *via* the condensation of the Vilsmeier–Haack reagent and amphoteric heterocyclic salts in the presence of anhydrous sodium acetate. The enhanced permeability, permeation, and retention (EPR) effect of the meso-chloride on a rigid cyclohexenyl ring of ZW800-Cl enabled ZW800-Cl to form a covalent complex with albumin and then penetrated the tumor cells *via* the overexpressed albumin receptors in cancer cells. ZW800-Cl exhibits intrinsic preferential tumor accumulation confirmed *in vivo* and *in vitro*. *In vivo* and *in vitro* photothermal experiments revealed that the ZW800-Cl solution and the tumor treated with ZW800-Cl showed a remarkable warming effect (74°Cat 30 s and 56°C at 3 min, respectively) compared with the control PBS group after irradiation with 808 nm laser, thereby confirming the excellent photothermal conversion property of ZW800-Cl. In addition, ZW800-Cl combined with the NIR laser can eliminate the tumor effectively with no toxicity during treatment and no recurrence within 7 days after treatment through a long-term phototherapeutic experiment in tumor-bearing mice (Figures 3F,G) (Lim et al., 2020).

However, as a side effect of PTT, the surging temperature causes cellular necrosis and then elicits pro-inflammatory response, and further triggers negative immune response, which may lead to tumor recurrence (Valcourt et al., 2019).

Danielle M. Valcourt et al. synthesized biodegradable polymeric NPs, comprising poly (lactic-co-glycolic acid) (PLGA) and loaded with IR820, to form monodisperse and spherical NPs (IR820-PLGA NPs) with a median hydrodynamic diameter of 60 nm. Flow cytometry analysis results showed that the cell binding and uptake of the NPs are dose- and time-dependent. IR820-PLGA NPs can promote apoptosis of TNBC and meanwhile have lower metabolic toxicity and stronger photothermal effect than the free IR820. In the mouse subcutaneous xenotransplantation model, the tumor volume of the free IR820 group was slightly reduced after treatment compared with that of the IR820-PLGA NP group and was significantly reduced or even completely disappeared under NIR laser irradiation (Valcourt et al., 2019).

### Photodynamic Therapy

**FIGURE 4:**
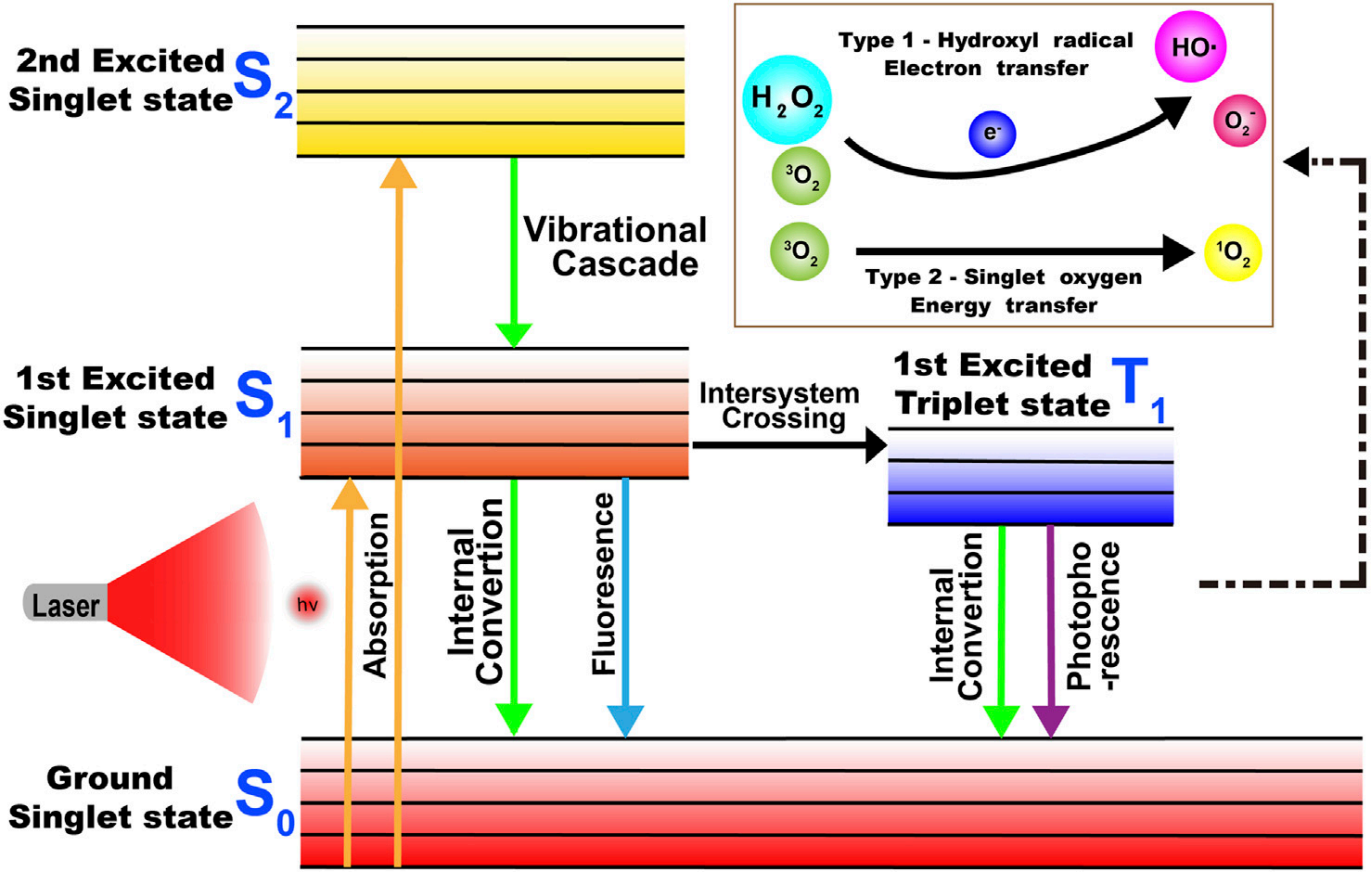
Mechanism of the production of ROS in PDT.

PDT, which exhibits non-invasive and unique spatial and temporal selectivity, has become a promising new type of cancer therapy (Yang G. et al., 2019; Zhao et al., 2019). In general, PDT depends on three essential components: light, oxygen, and photosensitizers (PSs) (Thomas et al., 2017; Zhao et al., 2019). When PS absorbs a specific wavelength of light, it is excited from the ground state to an excited singlet state and then returns to the ground state through non-radiative decay or fluorescence in a short period time, or it forms an excited triplet state which exists for a long time through system crossover and transfers light energy to the surrounding oxygen or loses electrons to produce cytotoxic ROS, inducing the apoptosis or necrosis of tumor cells by triggering oxidative stress or indirectly inhibiting tumor growth by destroying neovascularization (Figure 4). ROS reacts with protein thiol groups and lipids, resulting in cell membrane damage. Lipid peroxidation products easily induce the propagation of free radicals and hence, are destructive (Wilk et al., 2012; Thomas et al., 2017; Yang G. et al., 2019; Zhao et al., 2019). As the key element in PDT, an ideal photosensitizer should provide NIR absorption with high photostability, minimum dark toxicity, and high singlet–triplet system efficiency. Several Cy7 dyes and their derivatives have been studied as potential PDT dyes because of their excellent optical properties (Wilk et al., 2012; Shi et al., 2016; Thomas et al., 2017; Zhao et al., 2019).

IR783 is a commercially available NIR Cy7 dye with photodependent cytotoxic activity, similar absorption, and emission characteristics to ICG. In a study by Jordan Atchison et al., IR783 is modified by substituting iodine atoms for hydrogen atoms, and two iodized derivatives of IR783 (6a, 6b) were prepared. The experiment results showed that the singlet oxygen generation of 6a (7.9 fold, ^1^O_2_ = 0.66) and 6b (4.4 fold, ^1^O_2_ = 0.44) were significantly increased compared with that of ICG. The enhanced singlet oxygen generation for 6a relative to 6b is inspiring because of the remarkably reduced emission of the latter. In addition, a dose-dependent PDT-mediated increase in cytotoxicity was observed for 6a and 6b in two pancreatic cancer cell lines (BXPC-3 and MIA PaCa-2), and 6a was proved to be more potent than 6b in the MIA PaCa-2 cell line (Atchison et al., 2017).

A common strategy to improve the stability and targeting Cy7 is through structural modification and binding with specific ligands, such as TPP and hyaluronic acid (HA). The photodynamic therapeutic efficacy of the heptamethine dye combined with the cationic mitochondrial targeting agent can be improved. Ilkoo Noh et al. developed a mitochondria-targeting, brominated NIR fluorophore (MitDt-1) based on the cyanine dye as the PDT agent. This product is composed of brominated indolenine groups, a TPP moiety, and quaternary ammonium. MitDt-1 accumulates in the mitochondria of NCI-H460 and MCF-7 cancer cells *via* the TPP moiety. Meanwhile, the photochemical properties of the synthetic dyes showed that brominated dyes have high singlet oxygen productivity. In addition, ROS production is amplified for intensive and effective PDT under the on–off state of 662 nm single laser, resulting in mitochondrion destabilization apoptosis (Noh et al., 2018).

In a study by Ajesh P. Thomas et al., IR-Pyr, a Cy7 derivative, was developed by incorporating the pyridinium ion into the IR780 indocyanine skeleton. Compared with IR780, IR-Pyr exhibits a higher water solubility, better photostability, and preferential accumulation in the mitochondria. Electrostatic interactions between the negatively charged HA and the positively charged IR-Pyr were used to generate HA-IR-Pyr (Figure 3D). *In vitro* and *in vivo* PDT experiments showed that the generation ability of singlet oxygen by HA-IR-Pyr is better than that of IR-Pyr upon exposure to 808 nm laser irradiation (Figures 3E,H) (Thomas et al., 2017).

The tumor-specific microenvironment-activated PDT has been widely studied due to its efficient and precise therapeutic effect. In a study by Siriwalee Siriwibool et al., a pH-sensitive amino Cy7 based NIR probe (I_2_-IR783-Mpip) was developed for PDT and tumor imaging. In an acidic environment (pH = 5.0), I_2_-IR783-Mpip generated a high amount of ^1^O_2_ on exposure to 850 nm LED light for 30 min, and there was a good ROS probe generation in the cells. At concentrations as low as 20 μM, the probe exhibited photoinduced cytotoxicity to HepG2 cells with approximately 30 and 10% activity under physiological and acidic conditions, respectively. Moreover, I_2_-IR783-Mpip as a good tumor environment–targeting agent was able to maintain its PDT efficiency under a simulated deep-tissue setting (Siriwibool et al., 2020).

Guoliang Yang et al. synthesized a GSH-activatable pro-photosensitizer di-cyanine (DCy7) which is encapsulated in amphiphilic pH-responsive block copolymer POEGMA-b-PDPA to form P@DCy7 nanoparticles. Under non-reducing physiological conditions, these nanoparticles did not generate ROS upon 808 nm laser irradiation. However, after activation by intracellular GSH, P@DCy7 nanoparticles had high phototoxicity upon endocytosis of tumor cells. After intravenous injection, owing to the EPR effect, these nanoparticles can effectively accumulate in tumor tissues, deplete intracellular GSH, target tumor cells mitochondria, and achieve GSH-activation and efficient PDT (Yang G. et al., 2019).

Typically, the effect of Cy7s as photosensitizers is hampered by 1) poor water solubility; 2) a low molar extinction coefficient in the far-red region of light; 3) low production of singlet oxygen; and 4) non-targetability. Therefore, the limitations of PDT are still indispensable. (Atchison et al., 2017; Thomas et al., 2017).

### Photothermal Therapy and Photodynamic Therapy

Although the PTT and PDT of heptamethine and the derivatives as photosensitizers have potential for cancer therapy, any single phototherapy modality has limitations in large and deep vivo tumor therapy according to clinical observation due to the significant therapeutic effects decreasing over time (Luo et al., 2016; Lu et al., 2017; Cao et al., 2019; Xue et al., 2019). Compared with conventional single therapy, multimodal therapies integrating PDT and PTT, have been more widely applied in tumor treatment because they hybrid the merits of light-guided therapy, exhibiting relatively low toxicity, negligible metastasis, recurrence of tumor, and significantly improving the therapeutic effect (Luo et al., 2016; Lu et al., 2017; Xue et al., 2019).

Cao et al. modified Cy7 with heavy atom iodine to form the iodinated derivatives of Cy7, named CyI, which can simultaneously generate heat and enhanced ROS *via* the appropriate excitation of NIR light and maintain sufficient fluorescence signals for real-time imaging. *In vivo* and *in vitro* safety assessment showed the low cytotoxicity of CyI. CyI can also induce apoptosis in deep HepG2 tumors at higher inhibition rates than other non-iodinated NIR dyes, implying the considerable effect of tumor synergistic phototherapy (Cao et al., 2019). In another work of Jinnan Ch et al., a novel thermoluminescent nanocarrier HA-PEG-CyI (HPC) was easily prepared as an ideal immunomodulator for immunotherapy and synergistic phototherapy by inducing self-assembly of PEGylated CyI and connecting the ligand HA to the surface. HPC can accumulate in the tumor site owing to the EPR effect caused by its small size of 112.5 ± 1.8 nm and the increased active targeting ability from the modification of HA. *In vivo* and *in vitro* results showed that HPC can efficiently produce ROS and respond to high temperature under 808 nm laser irradiation, leading to the apoptosis and necrosis of the tumor. In addition, the tumor recurrence can be minimized due to a series of acute inflammatory reactions (Chi et al., 2020).

NGO-808 mentioned above also has great application prospects in tumor PDT/PTT combined treatment. The optimal laser wavelength (808 nm) of IR808 for PDT is consistent with that of NGO for PTT. NGO-808 produces a large amount of ROS and a high local temperature under 808 nm laser irradiation. Experiments have shown that the uptake efficiency of NGO-808 is higher than that of free IR-80 and strong hydrophobic IR-808. Evaluation of human and mouse tumor cells revealed that NGO-808 had significantly enhanced PTT and PDT effects compared with single PTT using NGO or PDT using IR-808 (Luo et al., 2016).

**FIGURE 5:**
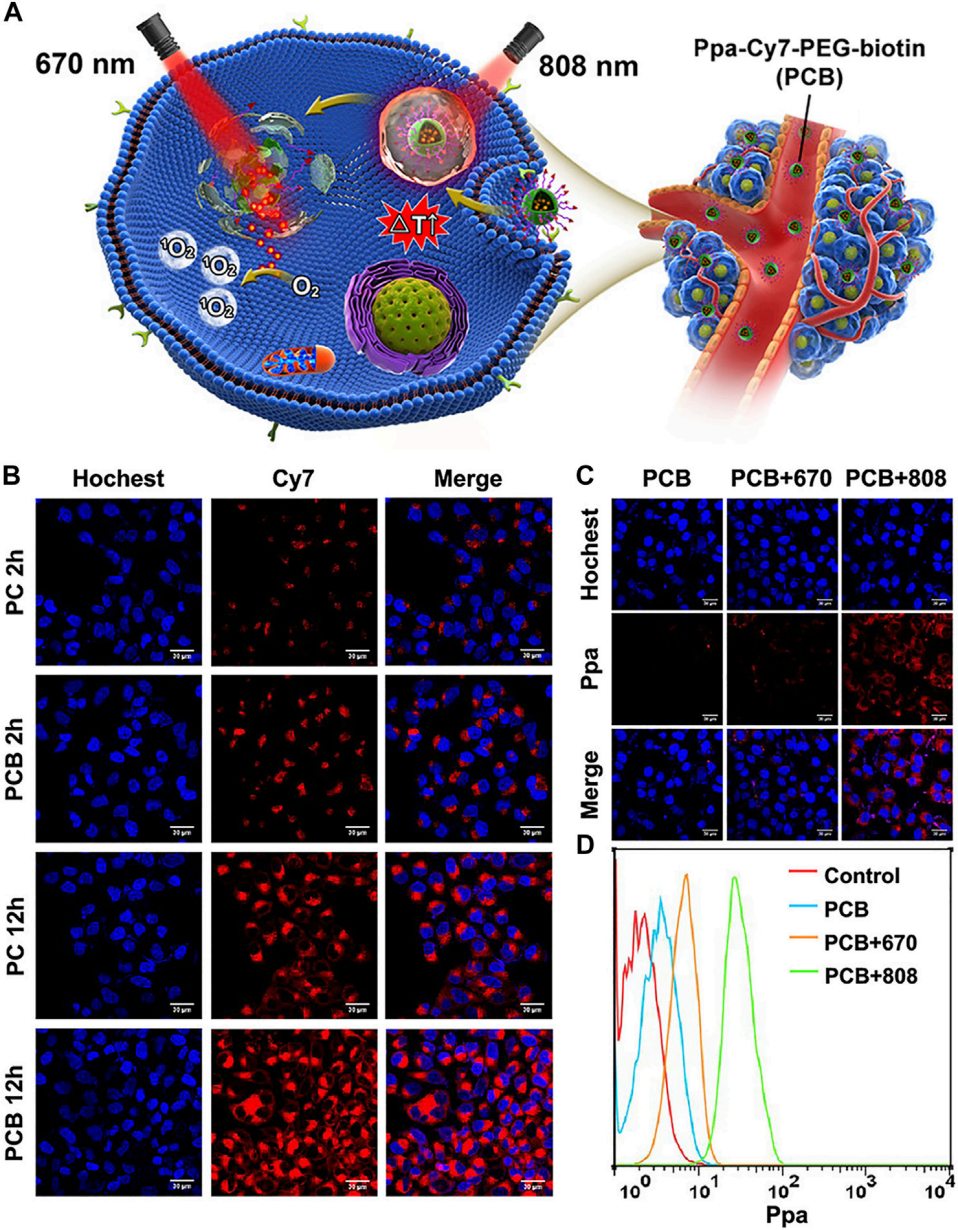
Application of PCB in tumor-combined phototherapy and characteristics of nanoparticles. Reproduced with permission from (Xue et al., 2019). **(A)**. Chemical structures and formation of Ppa-Cy7-PEG-biotin (PCB) and its application in NIR light activated multi-step phototherapy for the tumor. **(B)**. Confocal fluorescence images for comparing cell uptake of PC and PCB in HepG2 cells. Blue fluorescence is the cell nucleus stained with Hoechst; red fluorescence is the fluorescence of Cy7. **(C)**. Confocal fluorescence images for comparing intracellular release behavior of Ppa from PBC under different irradiations after incubation with PCB. Red fluorescence stands for the fluorescence of Ppa. **(D)**. Flow cytometry for analyze release and activation behavior of Ppa from PCB with irradiations of different wavelengths.

Unlike the generally combined phototherapy, a multistep phototherapy strategy based on photoactivation has great potential in cancer-enhanced combined phototherapy. In a study by Xue et al., Cy7 was used as a node to fabricate a NIR photodegradative nanomicelle (Ppa-Cy7-PEG-biotin, PCB) by covalently bonding it with pyropheophorbide A (Ppa) and PEG-biotin, to realize multistep PTT and PDT for the tumor. Under normal conditions, the photodynamic effect of the photosensitizer Ppa can be effectively inhibited by Cy7, but the photodegradable Cy7 enables it to be well-activated under 808 nm light irradiation when the micelles accumulate effectively in the tumor site *via* the EPR effect and active targeting (Figures 5A–D). In *in vivo* and *in vitro* IR thermal imaging, PCB micelles showed a surprising temperature rise effect. After intravenous injection of PCB micelles for 24 h, the temperature of the tumor site in HepG2 tumor-bearing mice rapidly increased from ≈37°C to ≈67°C within 5 min under 808 nm laser irradiation. In addition, compared with single PTT and PDT, multistep PTT and PDT achieved a more significant inhibitory effect on the growth of tumor cells, and the effect of PTT + PDT treatment was more pronounced than that of PDT + PTT (Xue et al., 2019).

Tony Duong et al. identified IR775, an efficient NIR hydrophobic ICG analog, that can achieve combinatorial phototherapy with a single phototherapy agent. In the work, IR775 was loaded into the nontoxic and biocompatible PEG−PCL polymeric nanoparticle. *In vitro* results showed that IR775-NP can efficiently generate both ROS and heat exposed to 785 nm laser irradiation, resulting in the eradication of cancer cells. Furthermore, animal studies exhibited that IR775-NP could efficiently accumulate in the tumor site after systemic administration, completely eradicating chemotherapy-resistant cancer tissues through combinatorial phototherapy (Duong et al., 2017).

## Heptamethine Cyanine7–Based Nanoprobes and Combination Therapy

At present, the occurrence and mortality rates of cancer are increasing. As traditional therapeutic agents, Cy7s inevitably exhibit limitations such as rapid photobleaching, poor pharmacokinetics, short blood circulation time, and possible cytotoxicity to normal cells (Bhattarai and Dai, 2017). Nanoparticle emergence has helped in overcoming the challenges mentioned above. Nanoparticles consist of nanocarriers and drugs loaded, unload medications in the presence of certain stimulation. Stimulation can be internal, such as pH, enzyme concentration, redox environment, or external, such as light, magnetism, and heat. (Tang Y. et al., 2018; Ye et al., 2018). Yang and co-workers synthesized a p (NIPAM-co-MACyanine-co-MCMEAM)-g-DOX with pH/NIR/heat-responsitivity to load DOX/MACyanine for cancer therapy (Yang R. et al., 2019). This indicates that modifying and assembling Cy7 dyes into nanoparticles, which can improve photophysical properties, photoconversion efficiency, ROS, hyperthermia, plasma half-life, targeting capability, bio-availability, and the bio-distribution of drugs, achieve combination therapy and multimodal imaging due to the unique characteristics, such as the small size, large volume ratio, adjustable surface chemistry, and the ability to encapsulate various drugs (Swain et al., 2016; Bhattarai and Dai, 2017; Gurunathan et al., 2018).

**FIGURE 6:**
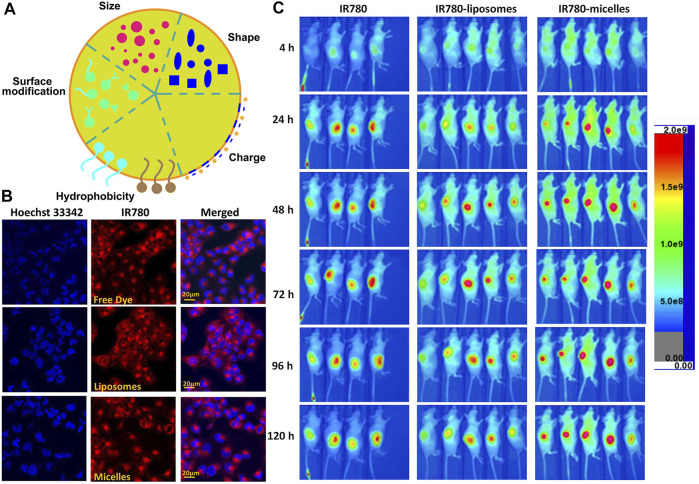
Characteristics of Cy7-based nanoparticles. **(A)**. Characteristics of nanoparticles. **(B)**. T98G cells were cultured with different formulations of IR780 for 30 min followed by Hoechst 33342 staining. Reproduced with permission from (Li S. et al., 2017). **(C)** *In vivo* NIRF imaging of IR780 nanoparticles with the U87M2/luc ectopic model. Reproduced with permission from (Li S. et al., 2017).

Innovative nanocarriers must possess more excellent drug-loading properties, cycle life, and optical and photophysical properties (Shi et al., 2016; Bhattarai and Dai, 2017). Various factors, such as the size of nanoparticles, hydrophobicity/hydrophilicity, surface charge, core components, and coating properties, affect the biodistribution of nanoparticles *in vivo* (Figure 6A) (DeFrates et al., 2018; Alphandéry, 2019). Adjusting the size of nanoparticles to a suitable size (the highest cellular uptake was observed at 10–60 nm) can increase the accumulation of nanoparticles in tumor sites, avoid the clearance of RES (reticuloendothelial system) organ, and prolong the half-life of blood circulation (Hoshyar et al., 2016). Studies on hydrophobicity/hydrophilicity have revealed that the PEG attached to the surface of nanoparticles inhibits the recognition and clearance of RES, thus prolonging the blood circulation time and changing the cell absorption mode and biological distribution (Verma and Stellacci, 2010; Bhattarai and Dai, 2017; Fornaguera and Solans, 2018). The surface charge and the counter ions around the nanosystem are the main factors that maintain the stability of the nanosystem and determine the biological distribution of nanoparticles to a great extent *in vivo* (Fornaguera and Solans, 2018; Neek et al., 2019). Cationic nanoparticles promote the depolarization of negatively charged cell membranes and increase the uptake of nanoparticles by cells, while anionic nanoparticles have a long blood circulation time (Bugno et al., 2016; Bhattarai and Dai, 2017). Moreover, spherical nanoparticles have higher cell uptake than other shapes (Neek et al., 2019).

Nanocarriers can be classified into three categories: 1) polymer-based nanocarriers, 2) lipid-based nanocarriers, and 3) inorganic nanoplatform composed of metal nanoparticles, silica nanoparticles, and carbon-based materials (Bhattarai and Dai, 2017). The heptamethine-based nanoparticles are elaborated in detail using this classification standard.

### Polymer-Based Nanocarriers

Polymers can be categorized as follows: 1) natural polymers, such as proteins, peptides, glycans, starches, and cellulose; 2) synthetic polymers, which are synthesized from natural monomers, for instance, polylactic acid (PLA) and PLGA; and 3) microbial fermentation polymers, such as polyhydroxybutyrate (Li Z. et al., 2017). It has been previously reported that nanoparticles are prepared by covalent bonding to the polymer backbone or chemically conjugated to the surface of the particles (Aryal et al., 2011). Mar í a Victoria Cano Cortes et al. designed three functional nanoparticles, namely, the chemotherapeutic drug DOX, NIR cyanine dye Cy7, and CRGDK homing peptide that specifically binds to neuropilin-1 (Nrp-1) overexpressed on triple-negative breast cancer (TNBC) cells. Compared with free DOX, nanoparticles improve the therapeutic effect, the first accumulation in the tumor area, and thus are able to be used in the treatment of triple-negative breast cancer (Cano-Cortes et al., 2020). Pan and his colleagues coupled the hydrophobic NIR cyanine IR825-NH_2_ with PEG-PLD *via* the amine-carboxyl reaction and then self-assembled to form PEG-PLD (IR825). This product increased the drug loading (∼21.0%), avoided the premature release of drugs to a great extent, and improved the anticancer effect. Owing to its sensitive fluorescence at ∼610 nm (552 nm excitation) and 830 nm (780 nm excitation), PEG-PLD (IR825) is suitable for fluorescence imaging *in vitro* and PTT guided by NIRF imaging *in vivo* (Pan et al., 2018). Xu et al. reported a nanoparticle Cy7-PEG-NP that enhanced the efficiency of photothermal conversion and could be used as a control agent for HeLa cells, MCF-7 cells, and mosquito larvae (Xu Q. et al., 2019). Further development is needed to co-package targeted components and NIR heptamethine into nanoparticles. Liu et al. prepared a novel dendrimer G5 NHAc-PEG-RGD/IR820 (a type of nanoparticle) by modifying G5 and an NH_2_ dendrimer with the RGD peptide, PEG chain, an acetyl group and then coating with IR820. RGD targets tumor cells by specifically binding with the α_v_β_3_ integrin on the surface of tumor cells, and the stability and biocompatibility are improved by using G5. The NH_2_ dendrimer acts as a carrier system and takes place in PEGylation (Liu and Wang, 2018). Similarly, chemotherapeutic drugs can also be co-packaged into nanoparticles to achieve phototherapy and chemotherapy combined treatment. Stimulus-responsive nanoparticles have been developed to achieve the precise trigger release of drugs. A novel pH/GSH dual-responsive nanoparticle P@DCy7 was prepared by Yang et al., in which two cyanines in DCy7 were covalently conjugated *via* a disulfide bond (activated by GSH). DCy7 was further encapsulated by the amphiphilic pH-responsive diblock copolymer POEGMA-b-PDPA (Yang G. et al., 2019).

Protein-based nanoparticles have attracted extensive attention because of their advantages, such as improving pharmacokinetics, achieving tissue targeting, promoting cell and tissue permeability, and biodegradability (DeFrates et al., 2018; Spicer et al., 2018; Neek et al., 2019). The most commonly used proteins are silk fibroin, keratin, collagen, gelatin, elastin, corn zein, and soy protein. However, casein, fibrinogen, hemoglobin, bovine serum albumin, and gluten have also been used to create nanoparticles (DeFrates et al., 2018). Gao and his colleagues designed and constructed a novel nanoparticle HSA/dc-825/GA through self-assembly of human serum albumin (HSA), dc-IR825, and gambogic acid (GA), which can cause the synergistic effect of mild PTT and chemotherapy to persistently and effectively ablate the tumor. Among them, GA blocks the overexpression of the heat shock protein HSP90 (Gao G. et al., 2019). To date, only a few reports were published about protein-based nanoparticles due to the high cost and possible immunogenicity (Bhattarai and Dai, 2017).

Polysaccharide (especially chitosan)-based nanoparticles, belong to another type of polymer-based nanoparticles. A novel nanoparticle MHI-HGC-PTX was designed and constructed based on HGC. MHI-148 is connected with HGC by a chemical bond, and PTX is embedded in it by the oil-in-water method, thereby forming MHI-HGC-PTX. Compared with free NIR dyes, MHI-HGC-PTX has higher accumulation in 4T1 tumors, indicating its enhanced tumor-targeting ability. MHI-148 combined with PTX achieves the synergistic effect of photothermal therapy and chemotherapy to improve the antitumor effect (Thomas et al., 2018). Zhang et al. modified chitosan with folate and Cy7 to form nanoparticle CF7Ns *via* self-assembly, and the folate was specifically binded to the folate receptor overexpressed on the surface of HeLa cells to achieve actively target HeLa cells. Compared with the non–folate-modified nanoparticles C7Ns, CF7Ns induced greater apoptosis of HeLa cells under NIR light irradiation (Zhang et al., 2017). Polysaccharide-based nanoparticles have broad prospects due to their simple and easy-to-obtain materials, excellent stability and biocompatibility, and potential tumor-targeting ability.

### Lipid-Based Nanocarriers

In polymer-based nanocarriers, drugs must be covalently encapsulated, which greatly limits the drug release. In contrast, liposomes have a hydrophilic internal structure, which makes the hydrophilic and hydrophobic drugs achieve synchronous delivery (Gao A. et al., 2019). The liposome is always the first choice of the small molecule drug delivery carrier (Ickenstein and Garidel, 2019). Liposomes have attracted extensive attention because of their enhanced pharmacokinetic properties, ease of manufacture in a size-controlled manner, and reduced non-targeted toxicity (Lee and Thompson, 2017; Ickenstein and Garidel, 2019). Julita Kulbacka et al. prepared a new type of SLNs by the solvent diffusion method, which was co-packaged with IR780, baicalein (BAI), or fisetin (FIS). The SLNs were stabilized by phospholipase 90G, and palmitate (CP) was used as the solid matrix. The results showed that SLNs increased the intracellular accumulation of drugs and improved the therapeutic effect (Kulbacka et al., 2016). To overcome the hydrophobicity of IR780, Li et al. prepared IR780 liposomes and IR780 phospholipid micelles. Both nanoparticles accumulated in the mitochondria and IR780 phospholipid micelles showed an enhanced tumor accumulation in U87MG heterotopic tumors and orthotopic glioma models, which provides a good prospect for clinical application in brain tumors (Figures 6B,C) (Li S. et al., 2017). To further increase the tumor accumulation of the drug and improve the antitumor effect, Xue and co-workers designed and constructed a new type of Adriamycin-conjugated carbon dots and indocyanine green–loaded liposome (CDs-ICG-LPs). Studies have shown that CDs-ICG-LPs have excellent fluorescence/dimensional stability, higher temperature response, and faster release of chemotherapeutic drugs under laser irradiation, achieving the synergistic effect of chemo-photothermal therapy and improving the therapeutic effect (Xue et al., 2018). As a NIR dye widely used in cancer treatment, ICG has a competitive relationship between imaging and treatment, and the hypoxic tumor environment further inhibits oxygen-dependent PDT, thus greatly limiting the application of ICG. Sheng et al. co-loaded perfluorooctyl bromide (PFOB) and ICG in liposome nanostructures to construct a new type of liposome nanoparticle LIP-PFOB-ICG that further realized CT imaging. The excellent oxygen-carrying capacity of PFOB compensates for the deficiency of oxygen-dependent PDT. LIP-PFOB-ICG realizes the synergistic treatment of PDT and PTT, improves the therapeutic effect, and could be a potential new reference in the application of cancer phototherapy (Sheng et al., 2018).

Stimulus-responsive liposomes accurately release loaded drugs under some kind of stimulation, such as pH, light, heat, and enzyme concentration, thereby preventing non-targeted losses and improving tumor drug accumulation. Xu et al. embedded ICG in a lipid bilayer and wrapped the water-soluble immunostimulatory molecule polyinosinic acid: polycytidylic acid (poly I: C) in a hydrophilic core, thus constructing the thermo-responsive liposome (piTRL). The results showed that the NIR laser irradiates ICG to generate heat, increasing the temperature of piTRL and inducing the release of poly I: C from liposomes in a temperature-dependent manner. Poly I: C further induces dendritic cell (DC) activation to achieve the photothermal immune synergistic therapy (Xu L. et al., 2019). In addition to the aforementioned liposomes and solid lipid nanoparticles, nanoemulsions and other forms have been explored. Although liposome-based nanoparticles have great advantages in application prospects, there is still a challenge in controlling and designing liposomes to enhance the antitumor effect.

### Inorganic Nanoplatforms

Inorganic nanoplatforms are generally divided into noble metals and their oxides, transition metal chalcogenides, and carbon-based materials with different dimensions, which tend to scatter light or convert into heat (Zhang P. et al., 2016). Inorganic nanomaterials have drawn attention due to their unique optical properties, magnetic properties, inertness, and various physical properties, such as fluorescence, NIR absorption, photoacoustic imaging, and magnetic resonance imaging. However, their long-term toxicity inevitably limits their further application because they are not easy to be removed from the body (Cherukula et al., 2016). Heptamethine-based inorganic nanocarriers are detailed in the next section.

Noble metals have strong surface plasmon resonance (Cherukula et al., 2016; Bhattarai and Dai, 2017), which greatly enhances the emission of light and results in better phototherapeutic properties than organic nanoparticles. Shriya S Srinivasan et al. loaded methoxyamine-modified cyanine-7 (Cy7MX) into polyethylene glycol–coated gold nanoparticles (AuNP) by binding Cy7MX with pyrimidine-/purine-free sites to inhibit the DNA repair mechanisms involved in cytotoxic chemotherapy (Srinivasan et al., 2019). Li and his colleagues assembled the Pt (IV) prodrug and cyanine dye cypate in the copolymer to overcome the challenges of cisplatin resistance and effectively ablating cisplatin-resistant tumors (Li et al., 2015). To overcome the problems of low light stability and photodegradation of IR820, Arpan Bera et al. bonded IR820 with ZnO nanoparticles to form IR820–ZnO nanohybrid. Analysis results showed that the light stability of IR820–ZnO was enhanced, and the ROS generation was detected, but the singlet oxygen was reduced. The use of the singlet oxygen quencher sodium azide confirmed that the singlet oxygen generation is directly related to the IR820 light stability (Bera et al., 2019). The transition metal chalcogenide nanoparticles have strong NIR absorption, and the absorbed energy is mainly converted into heat. Therefore, it is ideal for photothermotherapy (Zhang P. et al., 2016). However, there are limited reports on the co-packaging of transition metal sulfide and heptamethine into nanoparticles.

Mesoporous silica materials have received much attention because of their porous, biocompatibility, and high drug-loading capacities (Bhattarai and Dai, 2017; Nguyen et al., 2019). The large pore volume and surface area enable mesoporous silica nanomaterials to carry chemotherapeutic drugs, NIR cyanine dyes, and immune-stimulating molecules, which can achieve the synergistic treatment of photothermal, chemotherapy, and immunotherapy. At the same time, the surface is able to be coupled with targeted ligand molecules to realize the targeted release of drugs. For instance, Zhang et al. reported a novel multifunctional nanoparticle Cet-SLN/ICG based on mesoporous silica. Cetuximab coupled to the surface of silica nanoparticles targeting the overexpressed epidermal growth factor receptor in breast cancer. ICG was loaded in the pores of SLN to realize PTT for breast cancer. The results showed that Cet-SLN/ICG had better anti–breast cancer efficacy (Zhang et al., 2019b). Abhignyan nagesetti et al. reported a new silica nanoparticle system (Ormosil), which could load the chemotherapeutic drug DOX and heptamethine dye IR820. DOX was physically adsorbed or covalently linked to the silanol group to form FDSIR820 and CDSIR820, respectively, improving the effect of killing the tumor (Nagesetti and McGoron, 2016). To overcome the limitations of premature release of drugs and insufficient accumulation at tumor sites, Luping Sha et al. linked cypate to the surface of mesoporous silica nanoparticles *via* a disulfide bond and encapsulated d-α-tocopherol polyethylene glycol 1,000 succinate (TPGS) on the outer surface *via* the hydrophobic interaction to prevent drug leakage. Under the irradiation of the NIR laser, cypate produced heat and promoted lysosome rupture, thus inhibiting lysosome-mediated granodystrophy. TPGS also blocked the drug efflux, thus prolonging the drug action time and enhancing the antitumor activity (Sha et al., 2019).

In addition to the abovementioned types of nanoparticles, there are other innovative designs worthy of our reference. Xueluer Mu and other scholars made non-ionic heptamethine self-assembled into a 2D nano disk–like supramolecular structure, which had unique photothermal and photoacoustic properties and the characteristics of self-release and self-regulation (Mu et al., 2020). Wang et al. co-packaged covalent organic frameworks (COFs) with the chemotherapeutic drug cis-aconityl-doxorubicin (CAD) prodrug and heptamethine dye IR783 to form nanocomposites (COF@IR783@CAD), overcoming the limitations of low dispersion and water stability of COFs, prolonging the blood circulation time, and realizing the synergy of PTT and chemotherapy (Wang et al., 2019). Although how to adjust the nanoparticles to a reasonable property, greatly improve their stability, blood circulation time, photophysical properties, and increase the accumulation at tumor sites have not been detailed, heptamethine-based nanoparticles show great attractive characteristics compared with the single heptamethine cyanine.

### Combination Therapy

Tumors are difficult to completely ablate with the single drug treatment, which causes a series of problems such as drug resistance, recurrence, and metastasis. Combination therapy aims to load various drugs into drug delivery systems, especially nanoplatforms, release loaded drugs to tumor sites through active or passive methods, and achieve the synergy of multiple therapies. This technique reduces the use of toxic drugs and toxicity *in vivo*, improves the pharmacokinetic characteristics, and overcomes the challenges of drug resistance in conventional chemotherapy. Cyanine-based combination therapy, especially Cy7, mainly includes photochemical therapy, opto-electronic therapy, and photoimmunotherapy.

Photochemical therapy aims to encapsulate photosensitizers and chemotherapeutic drugs in the nanoplatforms. After being released at the tumor site, the photosensitizer produces heat or ROS under the irradiation of the NIR laser, killing the tumor together with the chemotherapeutic drugs to realize the synergy of photodynamic and chemotherapy or photothermal and chemotherapy. For example, p (NIPAM-co-MACyanine-co-MCMEAM)-g-DOX (Yang R. et al., 2019), HAS/dc-825/GA (Gao G. et al., 2019), MHI-HGC-PTX (Thomas et al., 2018), FDSIR820/CDSIR820 (Nagesetti and McGoron, 2016), and COF@IR783@CAD (Wang et al., 2019) mentioned above all realize the combination of PTT and chemotherapy and greatly improve the antitumor effect.

Electroporation is a simple way of increasing the drug transport (Kulbacka et al., 2016). Electrochemical therapy is a combination of electroporation and drug injection (Probst et al., 2018) that greatly promotes the intracellular accumulation of drugs and enhances the therapeutic effect. With the help of a nanoplatform, chemotherapeutic drugs can be encapsulated together with NIR cyanine dyes, especially Cy7, using electroporation technology to achieve the synergistic effect of electrochemical therapy and phototherapy. Packaging nanoparticles prolongs the blood circulation time in the body, increases the stability, and prevents premature drug leakage. The combination with electroporation further promotes the intracellular uptake of drugs, thus greatly increasing the drug action time, reducing the toxic drug dosage, and enhancing the antitumor treatment effect. It has been reported that heptamethine IR775 combined with electroporation can be used in drug-resistant breast cancer cells. The results showed that electroporation effectively transfers heptamethine IR775. The combination of electroporation and IR775-mediated PDT is hopeful to be used to treat drug-resistant cancer (Wezgowiec et al., 2018). Julita Kulbacha scholars mentioned above combined the SLNs containing IR780 with the electroporation technology, thus greatly improving the anticancer effect (Kulbacka et al., 2016).

Different from radiotherapy, chemotherapy, and surgery, immunotherapy for cancer indirectly kills tumors by restoring the power of the host immune system based on tumor immune escape (Li Z. et al., 2018). However, the nonspecific activation of the immune system damages the surrounding normal tissues, and low immunity is not sufficient to eliminate tumors. NIR photoimmunotherapy (NIR-PIT) is based on a combination of an antibody that can specifically bind to the antigen overexpressed on the tumor surface and a NIR dye. NIR-PIT initiates immunogenic cell death (ICD), simultaneously leaving the surrounding normal cells undamaged (Kobayashi and Choyke, 2019). Moreover, a photo-triggered cancer immunotherapy strategy based on NIR-responsive nanoparticles was developed and could trigger and activate killer T cells to kill tumor-related immune cells under NIR laser irradiation, thus inducing the immune evasion of cancer cells (Rajendrakumar et al., 2018). Chen et al. co-packaged R837, a Toll-like receptor agonist, and ICG in PLGA. Combining the immune response stimulation of R837 and the immune checkpoint blockade of CTLA4 caused an immune response that even inhibited metastases (Chen et al., 2016). At the same time, after the piTRL designed by Xu et al. mentioned above was irradiated by the NIR laser, ICG converted the light into heat and induced the release of immunostimulatory molecules poly I: C, thus further stimulating the immune response and realizing photothermal immune synergy therapy (Xu L. et al., 2019).

## Conclusion

We anticipate the development of non-invasive therapy modality. Cy7 exhibits dramatically better NIR absorption, fluorescence quantum yield, photoconversion efficiency, and other unique outstanding properties, compared with traditional cancer treatment such as surgery, chemotherapy, or radiotherapy, making diagnosis and therapeutics in one to realize real-time precise imaging-guided cancer phototherapy. In addition, Cy7-based derivatives and nanoparticles obtain further improved properties such as photophysics property, photostability, and extended blood circulation and simultaneously make combination therapy of cancer come true.

Undoubtedly remarkable preponderances have been displayed in Cy7-based NIRF imaging as well as theranostics. However, there is no doubt that Cy7s have the limitations of spatial resolution, penetration depth, and tissue scattering as the near-infrared fluorescence imaging agent. For single phototherapy modality, the development of Cy7s is hindered in large and deep vivo tumors therapy due to limited penetration depth. Other disadvantages, such as the poor hydrophobic property of IR780, the ineligible cytotoxicity of IR808, the high production cost for radionuclides-based cyanine dye, restrict the application and development in NIR imaging. Although innovative Cy7-based nanoparticles realize the control of drug loading and unloading, stretch blood cycle life, improve optical and photophysical properties, enhance targeting capability, and combine multimode therapy with multimodal imaging skillfully, how to accurately design and control innovative nanoparticles with suitable size and shape, better stability, proper surface charge, more compatible and stronger anticancer activity core, and excellent targeting shell is still an insurmountable problem. At the same time, it is undeniable that the premature release of loaded drugs, the high cost and possible immunogenicity of protein-based nanoparticles, and the long-term toxicity of inorganic nanoparticles due to the difficulty of being eliminated from the body have restricted the application of nanoparticles to a certain extent.

Therefore, we expect that developing novel Cy7-based derivatives and nanoparticles with optimized properties through chemical conjugation, covalent binding, or perfecting the preparation method. Similarly, finding out and designing novel cancer-targeting ligands to increase preferential accumulation and retention of anticancer drugs in tumor regions and mitigate nonspecific damage to surrounding tissues, which is also the objective numerous scholars are pursuing. It is also of extreme urgency that studying to realize imaging in deeper tissues as well as design and synthesize a novel multifunctional smart drug delivery system to preferably overcome limitations related to tumor recurrence and metastases.

Currently, some agents have gradually completed many clinical trials and come to the market, such as CY (Et)-Pan-DuoDM, FNIR-Tag-panitumumab, and CY (Me)-Pan-DuoDM, which strongly suggests the clinical practicability and feasibility of these emerging treatment modalities and agents. Furthermore, the translation to the clinical practice of agents based on Cy7 is also expected to resolve. In consequence, although obstacles remain, valuable and inspiring opportunities exist in the field of Cy7 in the foreseeable future.

## Glossary

Cy7: heptamethine cyanine
NIRF: near-infrared fluorescence
ICG: indocyanine green
NIR: near-infrared
PTT: photothermal therapy
ROS: reactive oxygen species
PDT: photodynamic therapy
TfR: transferrin-receptor
OI: optical imaging
CI: contrast index
CPP: cell-penetrating peptide
TMZ: temozolomide
TPP: triphenylphosphonium
HGC: hydrophobically modified glycol chitosan
PTX: paclitaxel
MRI: magnetic resonance imaging
FA: folate
PEG: polyethylene glycol: polyethylene glycol
BPEI: branched polyethylenimine
NGO: nanographene oxide
PAI: photoacoustic imaging
US: ultrasound imaging
CT: computed tomography
SPECT: single photon emission computed tomography
PET: positron emission tomography
GSH: glutathione
HES-SS-PTX: disulphide bond–linked hydroxyethyl starch paclitaxel
ACQ: aggregate fluorescence quenching
DTPA: diethylenetriaminepentaacetic acid
RGD: arginine–glycine–aspartic
PTAs: photothermal agents
SLN: solid liposome nanoparticle
AM: acrylamide
BisAA: N,N′-methylenebisacrylamide
DADMAC: diallyl dimethyl ammonium chloride
PLGA: poly (lactic-co-glycolic acid)
PSs: photosensitizers
HA: hyaluronic acid
Mpip: N-methylpiperazine
PEG: polyethylene glycol: polyethylene glycol
HPC: HA-PEG-CyI
Ppa: pyropheophorbide A
EPR: enhanced permeation and retention
PLA: polylactic acid
Nrp-1: neuropilin-1
TNBC: triple negative breast cancer
HAS: human serum albumin
GA: gambogic acid
BAI: baicalein
FIS: fisetin
CP: palmitate
PFOB: perfluorooctyl bromide
poly I:C: polyinosinic acid: polycytidylic acid
piTRL: thermo-responsive liposome
Cy7MX: methoxyamine-modified cyanine-7
AuNP: gold nanoparticles
TPGS: d-α-tocopherol polyethylene glycol 1,000 succinate
COFs: covalent organic frameworks
CAD: cis-aconityl-doxorubicin
NIR-PIT: NIR photoimmunotherapy
ICD: immunogenic cell death
